# Pilot Study of the Effect of Lanthanum Carbonate (Fosrenol®) In Patients with Calciphylaxis: A Wisconsin Network for Health Research (WiNHR) Study

**DOI:** 10.4172/2161-0959.1000162

**Published:** 2014-05-14

**Authors:** Micah R Chan, Fadi Ghandour, Narayana S. Murali, MJ Washburn, Brad C. Astor

**Affiliations:** 1 Division of Nephrology, Department of Internal Medicine, University of Wisconsin School of Medicine and Public Health-Madison, USA; 2 Division of Nephrology, Gundersen Health System, La Crosse, USA; 3 Division of Nephrology, Marshfield Clinic, Marshfield, USA; 4 Wisconsin Network for Health Research, University of Wisconsin School of Medicine and Public Health-Madison, USA; 5 Department of Population Health Sciences, University of Wisconsin School of Medicine and Public Health-Madison, USA

**Keywords:** Calcific uremic arteriolopathy, Calciphylaxis, Lanthanum

## Abstract

**Background:**

Currently there is a lack of effective treatment options for patients with calciphylaxis. There is anecdotal evidence that non-calcium based phosphorus binders may offer some benefit. The aim of this pilot study is to determine if lanthanum carbonate is effective in inducing remission of calciphylaxis lesions and demonstrate an improved DLQI (Dermatology Life Quality Index).

**Methods:**

This is a multi-site exploratory pilot study conducted through the Wisconsin Network for Health Research (WiNHR), a collaboration of health services researchers across the state of Wisconsin. Dialysis patients were recruited from in-center dialysis units, clinics and hospital admissions over a period of 24-months.

**Results:**

Due to the low inclusion rate, the trial was terminated after which 4 patients were prospectively analyzed. Dose of lanthanum carbonate was escalated to 3750 mg divided into 3 meals and titrated according to level of serum phosphorus. Gastrointestinal symptoms were the most common adverse effect. All 4 patients achieved complete remission by definition of skin re-epithelialization. Secondary outcome measurements showed a significant improvement in serum albumin (B coeff 0.17, 95% CI 0.002-0.031; p=0.023) and a significant improvement in overall DLQI score (B coeff -0.46, 95% CI -0.85- -0.08; p=0.019).

**Conclusions:**

Lanthanum carbonate appears to be efficacious as an adjunctive therapy to improve calciphylaxis lesions and symptom burden. More prospective clinical trials are warranted to determine the feasibility of this novel treatment strategy.

## Introduction

Calciphylaxis or calcific uremic arteriolopathy is an infrequently occurring, debilitating vasculopathy seen primarily in patients with end stage renal disease (ESRD) which almost always affects the skin. It has a prevalence rate of ~1-4% in long-term hemodialysis patients, with 1-year survival of 45% and an 8-fold risk of death as compared to the general dialysis population [1,2]. Despite being described in the literature for over 100 years, there has been no proven effective therapy. Lanthanum carbonate (FOSRENOL^®^) is a potent non-aluminum, non-calcium phosphate binder that was approved for use to reduce serum phosphate levels in patients with end stage renal disease. Since the proposed etiologic mechanism of injury and vascular calcification of calciphylaxis is predominantly hyperphosphatemia, elevated serum PTH, and hypercalcemia, lanthanum carbonate would be an ideal pharmacologic agent to utilize in this extremely enigmatic disease [3]. Furthermore, a recent case report demonstrated a significant improvement in laboratory parameters and calciphylaxis skin lesions with the use of lanthanum carbonate [4]. Our primary hypothesis is that since calciphylaxis represents the ultimate sequelae of metastatic vascular calcification predominantly involving hyperphosphatemia, elevated serum PTH, and hypercalcemia, lanthanum carbonate will be efficacious in its treatment.

Lanthanum carbonate or FOSRENOL^®^ is a potent phosphate binder that was approved for use by the FDA in October, 2004. It is indicated to reduce serum phosphate levels in patients with end stage renal disease. It is not metabolized and is not a substrate or inhibitor of CYP450. It inhibits intestinal absorption of phosphate by forming highly insoluble complexes, thereby lowering serum phosphorus and calcium-phosphorus product. Well designed, landmark studies have shown that doses of 375 mg/day up to a maximum of 2250 mg/day were effective in reducing phosphorus levels and calcium-phosphorus product as compared to placebo [5,6]. Adverse effects were predominantly gastrointestinal, including nausea, vomiting and abdominal pain but in a majority of cases well tolerated. Pre-clinical data has shown minimal systemic absorption and furthermore, bone biopsies in open-label active-controlled studies did not show differences in mineralization [5,7-8]. Two more recent studies have documented the efficacy and safety of lanthanum [9,10].

This is a preliminary open-label pilot study to test the ability of Wisconsin Network for Health Research (WiNHR) collaborative sites to collect information and data to determine if lanthanum carbonate will be efficacious in the treatment of calciphylaxis. The goal of the study is to:

- Determine if lanthanum carbonate is effective in eliciting remission (partial or complete) in calciphylaxis skin lesions

Secondary aims include:

- Evaluate the ability of each site to identify and enroll dialysis subjects with confirmed calciphylaxis into the study (a total of 12 subjects will be enrolled with non-UW sites enrolling approximately 4 per site)
- Correlate the relationship of lanthanum carbonate phosphorous control and calciphylaxis
- Evaluate additional information including laboratory parameters of intact PTH, calcium-phosphorous product levels, and albumin (as a marker of inflammation and nutritional status), as well as demonstrate an improved DLQI (Dermatology Life Quality Index) [11]. Preliminary data obtained in this study could guide the design of future prospective studies to evaluate the efficacy of lanthanum carbonate in this new indication.

## Methods

This pilot study (NCT01289626) had an intended enrollment of 12 patients and was approved by University of Wisconsin Health Sciences Institutional Review Board for all sites conducting the research. In addition, the FDA granted approval for the Initial New Drug application prior to initiation of the trial. This is a phase II clinical trial designed to evaluate efficacy. The principal investigator (MRC) had complete access to data acquired during the course of the pilot study and was responsible for the interpretation and analysis of the data and writing the manuscript. This study was performed from January 1, 2011, to December 31, 2012.

### Inclusion and exclusion criteria

Patients who were included in the study required the following criteria: (1) Participants will have provided informed consent; (2) Participants will be greater than or equal to 18 years of age; (3) Chronic renal failure receiving hemodialysis or peritoneal dialysis; (4) A diagnosis of calciphylaxis proven by skin biopsy or initial dermatology visit within the previous 5 years; and (5) Serum phosphorus > 4.5 mg/dL.

Patients were excluded from consideration if they had the following criteria: (1) Participants are not able to understand or provide written informed consent; (2) The research team deems that the participant may not be able to follow the study protocol; (3) Non-dependent HD patients receiving dialysis while in acute kidney injury; (4) Pregnant dialysis patient; (5) Active gastrointestinal obstruction or bleed; (5) Active inflammatory bowel disease; (6) Acute arteriovenous graft occlusion; (6) Known hypersensitivity to lanthanum carbonate

This study was conducted following all local laws and regulations in the conduct of research, as well as the International Conference on Harmonisation’s Good Clinical Practice Guidelines. These guidelines provide a unified standard for designing, conducting, recording, and reporting trials that involve the participation of human subjects.

## Study Design

This is an open-label pilot study to assess the efficacy of lanthanum carbonate in patients with calciphylaxis divided into 2 phases; a washout period of 4-weeks if patient was previously on lanthanum carbonate and a treatment period lasting 12-weeks.

All patients had a baseline physical examination, a recent history of dermatology consult, and photograph of calciphylaxis involved skin lesions. Laboratory parameters included intact PTH, phosphorus, calcium, and albumin (all of which are routinely measured in dialysis patients).

All long-term medications patients received were documented. Lanthanum carbonate was administered orally in a dose of 1500- 3750 mg daily in divided doses with meals. Dose escalation was adjusted carefully to a target dose of 3750 mg daily or as designated by clinician over a 4-week period from start of study. The drug was administered for 12 weeks. This dose was increased over 4-weeks to 3750 mg total in divided doses (1250 mg three times with meals) to keep phosphorus levels between 3.5-5.5 mg/dl. The clinician increased the dose from 500 mg to 750 mg the second week, 750 mg to 1000 mg the third week and finally 1250 mg the fourth week. The level of phosphorus was measured at the second month as part of study protocol. If the phosphorus level was below 3.5 mg/dl, then the dosage was decreased by 20% to 3000 mg daily in divided doses. If the phosphorus level was still below 3.5 mg/dl at the third month, the dose was decreased by 25% to 2250 mg daily in divided doses. If the phosphorus level was still below 3.5 mg/dl at the fourth month of the study, the dose was decreased by 33% to 1500 mg daily in divided doses. If levels of phosphorus continued to be elevated above 5.5 mg/dl, then the clinician could add non-calcium based binders of choice according to standard clinical practice. The study drug is an established and widely prescribed drug currently in dialysis patients. Guidelines for drug inventory and management was established by the University of Wisconsin Pharmaceutical Research Center and utilized at all study sites. The drug is used in standard practice and clinicians are accustomed to increasing dosages.

Patients were evaluated monthly to determine clinical response to treatment during a period of 3 months. A final photograph was taken to document response to therapy at the conclusion of the treatment period. The subject then completed a final telephone follow-up four weeks after treatment discontinuation. We reviewed all adverse events for 30 days post-last dose of study drug (Table 1).

Twelve patients total were intended to be enrolled through WiNHR sites (University of Wisconsin and Affiliated Hospitals, Wisconsin Dialysis Inc.; Marshfield Clinic; and Gundersen Lutheran. Approximately 4 patients from each non-UW site were expected for recruitment (Table 1).

## Measures

Response to therapy was defined as clinical response (i.e., complete or partial remission) of skin lesions to medication. Remission was based on a reduction in ulcer size and depth as well as an assessment of the degree of undermining of the ulcer edge. Complete wound closure and therefore complete remission was defined as skin re-epithelialization without drainage or dressing requirements confirmed at two consecutive study visits as cited in the FDA’s Guidance for Industry Chronic Cutaneous Ulcer and Burn Wounds — Developing Products for Treatment booklet. Partial remission was defined as skin re-epithelialization that still exhibits drainage or dressings. We expected at least a 50% complete remission (CR) or partial remission (PR) of skin lesions to consider the treatment effective.

The primary driving force for vascular calcification is elevated calcium and phosphorus which were measured as outcomes. We expected that since phosphorus control would improve based on efficacy data from multiple clinical trials, an improvement in secondary hyperparathyroidism and increased albumin levels should also improve as a result of improvement in overall health, nutritional and inflammatory status. We anticipate that there will be an improvement in quality of life compared to historical data. The DLQI survey is validated for dermatological conditions such as calciphylaxis (Table 2).

## Statistical Methods

Sample size and power were calculated based on number of recruited subjects that were expected to achieve CR or PR. A sample size of 12 was required under the assumption of 50% of patients having CR or PR, such that the lower 95% confidence level would extend to 0.283. Mixed effect linear regression models were used to examine the changes in DLQI variables associated with lanthanum treatment, while taking into account the within-person correlations. We also examined which laboratory values were affected by the treatment of lanthanum, controlling for baseline variables. For all statistical tests, two-sided alpha levels were set a priori at 0.05. The statistical analyses were performed using Stata MP 12.1.

## Results

Five patients were enrolled in the study during the 24-months of recruitment but only four completed the entire treatment period of 12-weeks. All four patients were women and ranged in age from 32 to 74 years (mean 51.8 ± 17.3). The ethnicity of all patients was white. There was a variety of causes of ESRD including IgA nephropathy, diabetes mellitus, systemic lupus erythematosus and hypertension. These patients used a variety of medications for mineral bone disorders, including sevelamer carbonate, sevelamer hydrochloride, calcium acetate and cinacalcet, however per protocol, dosages did not change during treatment period. Caloric intake and nutritional status did not change during treatment period. There were no serious adverse events. The most common side effects were gastrointestinal and musculoskeletal occurring in two patients.

## Primary Outcome

**Figure 1:**
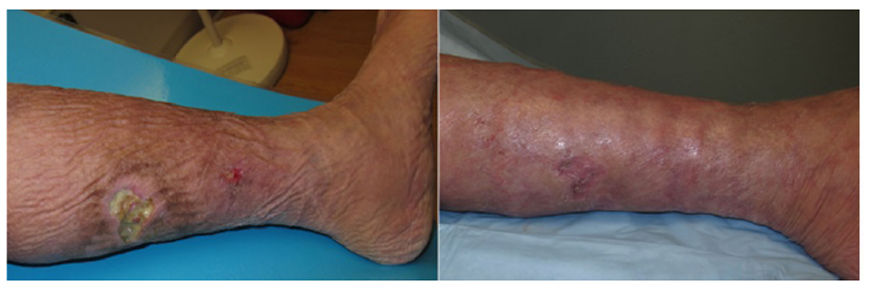
Before (left) and after (right) pictures of ulcerative calciphylaxis lesion after 12-week treatment with lanthanum carbonate.

**Figure 2:**
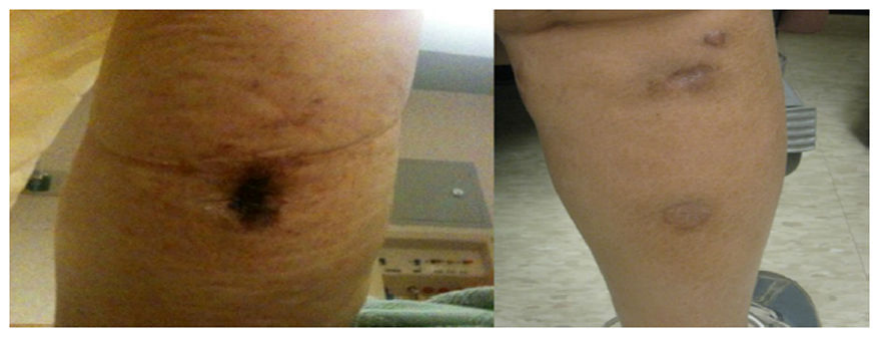
Before (left) and after (right) treatment of early calciphylaxis skin lesions after 12-weeks of lanthanum carbonate.

The primary outcome of complete remission of calciphylaxis skin lesions was seen in all four patients enrolled in the study. Before and after photos of skin lesions are shown in two patients in different stages of lesion progression (Figure 1 and 2). Complete wound closure and therefore complete remission as defined as skin re-epithelialization without drainage or dressing requirements was confirmed at two consecutive study visits in all patients.

## Quality of Life

The DLQI is calculated by summing the score of each question (survey of 10 questions) resulting in a maximum of 30 and a minimum of 0. The higher the score, the more quality of life is impaired. The DLQI can also be expressed as a percentage of the maximum possible score of 30. Meaning of DLQI Scores:

0-1 = no effect at all on patient’s life
2-5 = small effect on patient’s life
6-10 = moderate effect on patient’s life
11-20 = very large effect on patient’s life
21-30 = extremely large effect on patient’s life

The DLQI questionnaire showed an improvement in median scores after patients received treatment with lanthanum carbonate as demonstrated in Table 3, specifically in symptoms and feelings (p=0.01), personal relationships (p=0.03), treatment effect (p=0.03) and overall score (p=0.02).

Data are expressed as standardized regression coefficient (β) with 95% Confidence Intervals and P-values.

### Laboratory parameters

Laboratory data at the end of the 12-week treatment period for study subjects are listed in Table 4. None of the patients had significant derangement of their basic metabolic panels during the treatment phase. There was a consistent improvement in serum albumin levels throughout treatment with lanthanum carbonate with an average increase in 0.012 g/dL per day (p=0.02). Serum calcium, phosphorus, and intact parathyroid hormone did not change significantly with the use of lanthanum carbonate.

Data are expressed as standardized regression coefficient (β) with 95% Confidence Intervals and P-values.

## Discussion

To date, this is the first open-label pilot study to assess the efficacy of treating calciphylaxis in patients with ESRD. Even though the disease process has been described since 1898, the pathophysiology and treatment remains elusive [12,13]. We reported a severe case of calciphylaxis in a patient with acute kidney injury and liver disease who responded rapidly with lanthanum carbonate over a period of eight weeks [4]. Given one of the proposed etiologic mechanisms of injury and vascular calcification of calciphylaxis is elevated serum PTH, hypercalcemia, and hyperphosphatemia, it made sense to us that lanthanum improved the clinical status by directly affecting these laboratory values. However, there have been many other ways of achieving the same effects, ie. low-calcium dialysate, parathyroidectomy, calcimimetics, bisphosphonates, hyperbaric oxygen and intravenous sodium thiosulfate which do not uniformly improve the calciphylaxis skin lesions [14-18]. To that end, we believe that lanthanum carbonate not only improves secondary hyperparathyroidism and hyperphosphatemia, but also has pleiotropic effects not well understood. In fact, it has been recently shown that lanthanum carbonate effectively lowers FGF-23, urinary phosphate, improves arterial medial calcification (AMC) and may improve nutritional status of dialysis patients [19-21]. The predominant theory of calciphylaxis is that it is a systemic syndrome which almost always affects the skin with characteristic calcification involving the media of small to medium-sized arterioles. This is characterized by the Von kossa stain which targets calcium deposition in the vessels and is diagnostic for calciphylaxis on skin biopsy.

Our results showed for the first time an improvement in skin lesions documenting complete remission over a 12-week treatment period. The patients also showed an improvement in the DLQI questionnaire which is a validated tool in dermatologic studies. The intact PTH, phosphorus and calcium did not change significantly during the trial likely due to the short duration of treatment. However, serum albumin levels significantly improved during the trial. Higher serum albumin is a surrogate marker of improved nutrition and possibly reduced inflammatory status.

There were certain limitations to this study given its observational nature, including the inability to reach original target sample size, patients’ demographic factors, hemodynamic status, and medications. These confounding factors may all play critical roles in the pathogenesis and pathology of calciphylaxis.

In conclusion, it is feasible to study the effect of short-term treatment with lanthanum carbonate on dialysis patients with calciphylaxis. In view of the proven safety and tolerability of this medication in this pilot study and many others, we propose that a multicenter randomized, placebo-controlled trial be performed to determine the safety and efficacy of this potentially novel therapeutic regimen for dialysis patients with calciphylaxis.

## Figures and Tables

**Table 1:** Baseline characteristics of patients

Patient	Age	Sex	Cause of ESRD	Use of other phosphorus binding agents	Adverse Events
1	74	F	Hypertension	Phoslo, renvela	gastrointestinal
2	49	F	Hypertension	Renagel	n/a
3	32	F	IgA nephropathy	Renvela	n/a
4	52	F	Lupus	n/a	musculoskeletal

**Table 2:** Dermatology Life Quality Index Score (DLQI)

Variables	Question Number	Score Range
Symptoms and feelings	Questions 1 and 2	0-6
Daily activities	Questions 3 and 4	0-6
Leisure	Questions 5 and 6	0-6
Work and School	Question 7	0-3
Personal relationships	Questions 8 and 9	0-6
Treatment	Question 10	0-3

**Table 3:** Change in DLQI scores (per day) with lanthanum carbonate treatment.

Variable	β	95% CI	P-value
Overall score	-0.46	(-0.85 to -0.078)	0.02
Symptoms and feelings	-0.067	(-0.12 to -0.013)	0.01
Daily Activities	-0.095	(-0.21 to -0.023)	0.12
Leisure	-0.091	(-0.19 to 0.005)	0.06
Work and school	-0.035	(-0.08 to 0.009)	0.12
Personal relationships	-0.121	(-0.23 to -0.012)	0.03
Treatment	-0.056	(-0.11 to -0.005)	0.03

**Table 4:** Change in laboratory paramters (per day) with lanthanum carbonate treatment.

Variable	β	95% CI	P-value
Albumin (g/dL)	0.017	(0.002 to 0.031)	0.02
Serum calcium (mg/dL)	0.003	(-0.04 to -0.047)	0.88
Serum phosphorus (mg/dL)	-0.095	(-0.11 to 0.085)	0.80
Intact PTH (pg/mL)	0.94	(-3.77 to 5.66)	0.70
